# APIM-Mediated REV3L–PCNA Interaction Important for Error Free TLS Over UV-Induced DNA Lesions in Human Cells

**DOI:** 10.3390/ijms20010100

**Published:** 2018-12-28

**Authors:** Synnøve Brandt Ræder, Anala Nepal, Karine Øian Bjørås, Mareike Seelinger, Rønnaug Steen Kolve, Aina Nedal, Rebekka Müller, Marit Otterlei

**Affiliations:** 1 Department of Clinical and Molecular Medicine, Faculty of Medicine and Health Sciences, Norwegian University of Science and Technology (NTNU), N-7491 Trondheim, Norway; synnove.b.rader@ntnu.no (S.B.R.); anala.nepal@ntnu.no (A.N.); karine.bjoras@ntnu.no (K.Ø.B.); mareike.seelinger@ntnu.no (M.S.); ronnaugsk@gmail.com (R.S.K.); ainanedal11@gmail.com (A.N.); rebrekka.muller.phd@gmail.com (R.M.); 2 Clinic of Surgery, St. Olavs Hospital, Trondheim University Hospital, N-7006 Trondheim, Norway

**Keywords:** POLζ, mutation frequency, mutations spectra, SupF, mutagenicity

## Abstract

Proliferating cell nuclear antigen (PCNA) is essential for the organization of DNA replication and the bypass of DNA lesions via translesion synthesis (TLS). TLS is mediated by specialized DNA polymerases, which all interact, directly or indirectly, with PCNA. How interactions between the TLS polymerases and PCNA affects TLS specificity and/or coordination is not fully understood. Here we show that the catalytic subunit of the essential mammalian TLS polymerase POLζ, REV3L, contains a functional AlkB homolog 2 PCNA interacting motif, APIM. APIM from REV3L fused to YFP, and full-length REV3L-YFP colocalizes with PCNA in replication foci. Colocalization of REV3L-YFP with PCNA is strongly reduced when an APIM-CFP construct is overexpressed. We also found that overexpression of full-length REV3L with mutated APIM leads to significantly altered mutation frequencies and mutation spectra, when compared to overexpression of full-length REV3L wild-type (WT) protein in multiple cell lines. Altogether, these data suggest that APIM is a functional PCNA-interacting motif in REV3L, and that the APIM-mediated PCNA interaction is important for the function and specificity of POLζ in TLS. Finally, a PCNA-targeting cell-penetrating peptide, containing APIM, reduced the mutation frequencies and changed the mutation spectra in several cell lines, suggesting that efficient TLS requires coordination mediated by interactions with PCNA.

## 1. Introduction

DNA damage is continuously induced by exogenous and endogenous sources. If not repaired prior to replication, these may result in replication fork collapse, strand breaks, cell death, or genomic instability. Cells have; therefore, evolved fine-tuned systems to handle replication fork stalling via two main pathways: translesion DNA synthesis (TLS) and template switching (TS). TLS is intrinsically error-prone and a major source of mutations, while TS is mostly error-free [1].

Proliferating cell nuclear antigen (PCNA) belongs to the conserved DNA clamp family, and the earliest known function of PCNA was docking of replicative polymerases to DNA. PCNA is a hub protein and essential for multiple DNA replication-associated processes, for example, chromatin remodeling/epigenetics, DNA repair, recombination/TS, and TLS [2,3]. When the replication fork encounters a DNA lesion, mono-ubiquitination of PCNA is suggested to be important for mediating a polymerase switch, from the replicative polymerase to a TLS polymerase, which is able to synthesize over the lesion. In addition to the polymerase switch at replication forks, TLS polymerases are also believed to be important for the filling of post-replicative gaps left by replicative polymerases [1].

Several hundred proteins contain one or two of the PCNA-interacting motifs, called PCNA interacting peptide (PIP)-box and AlkB homolog 2 PCNA interacting motif (APIM), both of which are conserved in yeast [4,5]. These PCNA-binding motifs have an overlapping interaction site on PCNA [6,7,8,9]. The selection of which proteins interact with PCNA at any given time is likely coordinated by multi-layered regulatory mechanisms, including affinity-driven competition, post translational modifications (PTMs) of PCNA or PCNA-binding proteins, complex partners, as well as translational and proteolytic regulations [2].

The main polymerases in TLS are the Y-family polymerases, REV1, POLη, POLι POLκ and the B-family polymerase, POLζ. POLζ is an extender polymerase (i.e., it extends from the mismatch generated by the “inserter” TLS polymerases, POLη, POLι, or POLκ. However, POLζ has also been shown to insert bases opposite lesions [1]. The POLζ complex (here called POLζ consists of four subunits; REV3L, REV7, p50 (POLD2), and p66 (POLD3) [10]. The latter two are shared with the lagging strand replicative polymerase, POLδ. REV3L was recently also shown to be localized in mitochondria, where it associated with POLγ [11]. REV3L, the catalytic subunit, is essential for development and survival, for example, embryonic lethality is observed after REV3L knock out (KO) in mice [12,13], and higher sensitivity to UVC irradiation and chemotherapeutics, such as mitomycin-C (MMC), is seen in human cells with catalytically dead REV3L and REV3L KO cells [14]. The mammalian REV3L is 3130 amino acids (aa) long, which is twice the size of its yeast ortholog [12], and it contains the PCNA-interacting motif APIM in the predicted unstructured region (PCD) (aa 1240–1244), which is not present in yeast [5,15]. Whether APIM in mammalian REV3L is functional is not known.

REV1 acts as a scaffold for TLS via interactions with POLη, POLι, POLκ, POLζ subunit REV7, and PCNA. It is suggested that REV1 interacts with PCNA via its N-terminus BRCA1 C terminus (B RCT) domain and/or polymerase-associated (PAD) domain. POLη, POLι and POLκ contain the PIP-box, and interact directly with PCNA [1], but still they are dependent on REV1 for replicating over UV lesions [16]. POLζ, which contains a potential APIM in REV3L, can replicate over UV lesions independently of REV1. How exactly the most appropriate TLS polymerase is selected when needed likely depends both on the type of DNA lesion and on their ability to interact with their two main hub proteins, REV1 and PCNA.

In this study, we examined the in vivo properties of overexpressed full-length REV3L, and the functionality of APIM found in the predicted unstructured region of REV3L. Colocalization experiments, as well as analysis of mutation frequencies and mutations spectra after overexpression of full-length REV3L, supports that APIM in REV3L, and its direct interaction with PCNA, is important for REV3L’s function in TLS. Furthermore, we found that an APIM-containing cell-penetrating peptide (APIM-peptide) targeting PCNA [6,17] reduced the mutation frequency more in the isogenic normal cell line than in POLζ-mutated cells. This data supports a role of APIM–PCNA interactions in TLS, and specifically in POLζ-mediated TLS.

## 2. Results and Discussion

### 2.1. REV3L Localization Increases in the Nuclei upon Genotoxic Stress and Inhibition of Nuclear Export

**Figure 1:**
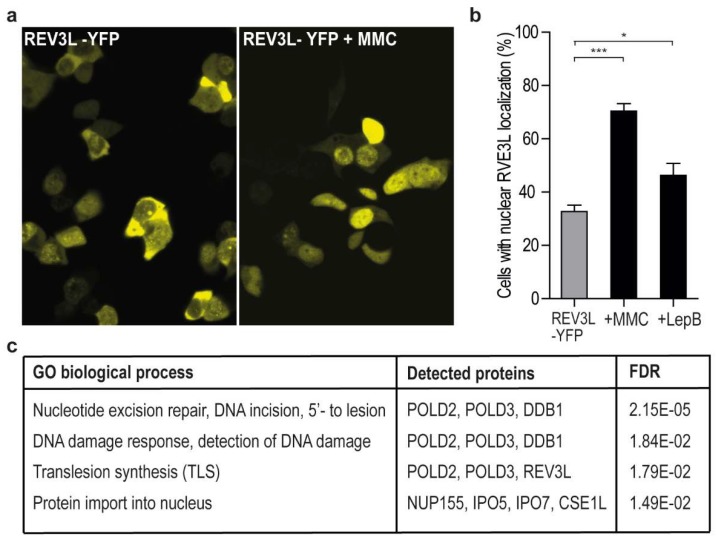
Increased nuclear REV3L localization after mitomycin-C (MMC) or Leptomycin B treatment. (**a**) Overview of subcellular localization of REV3L-YFP in HEK293T cells with and without MMC treatment (0.5 µM), measured after 12 h treatment. (**b**) Quantification of REV3L-YFP nuclear localization with and without MMC (0.5 µM) and Leptomycin B (LepB; 10 ng/mL, 1 h) treatment. Data from three independent biological experiments, each counting a minimum of 150 cells. (Student *t*-test, * *p* = 0.017, *** *p* < 0.0001). (**c**) Gene ontology (GO) analysis of proteins co-immunoprecipitated with REV3L-YFP using a PANTHER overrepresentation test. GO biological processes with a Benferroni-corrected *p*-value < 0.05 are shown. p-values given as false discovery rate (FDR).

To determine REV3L localization, we overexpressed full-length REV3L tagged with YFP. REV3L localized both in the nucleus and cytosol, but the fraction of cells with nuclear localization increased after MMC treatment and UV irradiation (Figure 1a,b, and data not shown). This is in accordance with increased chromatin association after genotoxic stress previously reported [18]. Recently, REV3L was found to contain both functional nuclear and mitochondrial localization signals, and to associate with POLγ in mitochondria [11]. Some of the cytosolic REV3L could; therefore, be mitochondrial, but it could also be due to overexpression. However, lack of specific antibodies against REV3L makes this hard to examine. Cytosolic localization could also indicate that the level of REV3L in nuclei is tightly regulated, for example by active nuclear export followed by protein degradation, to avoid mutagenic events. When the cells were treated with a specific inhibitor of active nuclear export, Leptomycin B [19], the fraction of cells with mainly nuclear REV3L-YFP localization increased (Figure 1b). This was not seen in cells expressing only the YFP-tag, where no cells had nuclear localization, even after Leptomycin B treatment (Supplementary Figure S1a,b). These results support that nuclear levels of REV3L are regulated via active nuclear export.

In order to further characterize REV3L and its interaction partners*,* pull down experiments, using an anti-YFP antibody on extracts from weakly cross-linked cells overexpressing REV3L-YFP or YFP only, were performed. A weak PCNA band was detected on western blots after immunoprecipitation (IP) with anti-GFP from REV3L-YFP expressing cells, but also in some IPs from the control cells (YFP expressing cells). Thus, it was hard to determine if PCNA pull downs were significantly enriched in the REV3L-pull downs (data not shown). Next, we analyzed the same samples by mass spectrometry (MS). We did not detect PCNA, likely because PCNA is a “bad” flyer and not easily detected by MS [20]. However, we detected numerous proteins specifically pulled down by REV3L, including the subunits shared with POLδ, POLD2, and POLD3 (Figure 1c and Supplementary Table S1a). This suggests that the overexpressed tagged REV3L is functional and in complex with its normal partners. Gene ontology (GO) analysis revealed that the potential REV3L interaction partners were associated with the following biological processes: nucleotide excision repair, DNA damage response, detection of DNA damage, protein import into nucleus and TLS (Figure 1c). We filtered proteins using the “CRAPome” database (www.crapome.org) prior to this analysis, however the biological processes detected did not change much from the list, including all proteins regarded as significantly enriched in REV3L-YFP pull downs (Supplementary Table S1b).

### 2.2. REV3L Colocalizes with PCNA and Contains a Functional APIM Sequence

**Figure 2:**
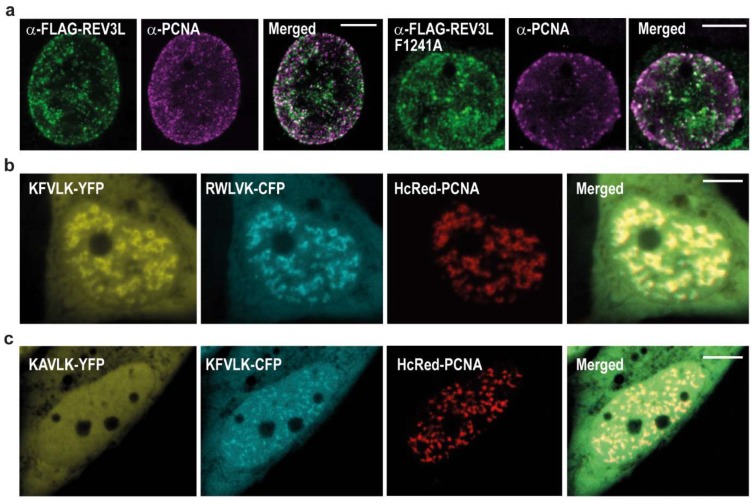
Overexpressed full-length REV3L and APIM peptide from REV3L (KFVLK-YFP) colocalizes with endogenous and overexpressed PCNA. White line on merged images represents 5 µm scale. Representative images display: (**a**) REV3L or REV3L F1241A (α-FLAG) and endogenous PCNA (α-PCNA) in transfected HEK293T cells (STED image); (**b**) KFVLK-YFP (REV3L APIM), RWLVK-CFP, and HcRed-PCNA in transfected HeLa cells (live cell images); and (**c**) KAVLK-YFP (REV3L F1241A-APIM), KFVLK-CFP (REV3L APIM), and HcRed-PCNA in transfected HeLa cells (live cell image).

The four subunit yeast POLζ complex is reported to have higher activity in presence of PCNA [10], thus how the different subunits interact with PCNA may be important, both for proper regulation of their activity and possibly also for fidelity/substrate specificity. The POLζ complex has two PCNA interacting motifs, POLD3 contains a PIP-box and REV3L contains the APIM sequence KFVLK (1240-1244). Previous data has shown that the second amino acid in APIM (consensus sequence: R/K-F/W/Y-L/I/V/A-L/I/V/A-K/R) is vital for affinity to PCNA [5,6,17,21]. After mutation of this amino acid, we found that both REV3L and REV3L F1241A colocalized in PCNA foci resembling replication foci (Figure 2a). This was not unexpected since REV3L pulled down both POLD2 and POLD3, both having the ability to interact directly or indirectly with PCNA [22,23,24]. Therefore, reducing the APIM-mediated REV3L–PCNA interaction by the F1241A mutation might not be sufficient to abolish colocalization with PCNA. However, KFVLK is a functional PCNA interacting motif, as KFVLK-YFP colocalizes with HcRed-PCNA in foci resembling replication foci (Figure 2b), similarly to the previously reported hABH2_1–7_F4W APIM-variant (RWLVK) with increased affinity [5] (Supplementary Figure S1c), here shown as a CFP-fusion. Furthermore, when F in KFVLK is mutated (corresponds to F1241A in REV3L), colocalization with PCNA is strongly reduced (Figure 2c).

**Figure 3:**
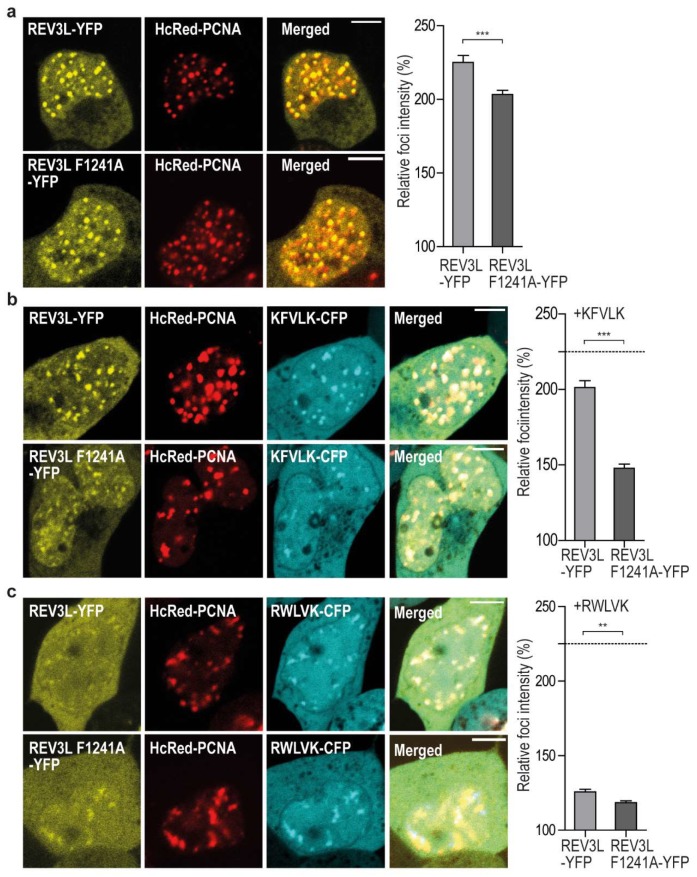
REV3L colocalization with PCNA in replication foci is reduced upon F1241A mutation and overexpression of APIM-peptides. Left panel: Representative live cell images of HEK293T cells overexpressing REV3L-YFP (upper row) or REV3L F1241A-YFP (lower row) and HcRed-PCNA. Scale bar = 5 µm. Right panel: Quantification of foci intensities of REV3L-YFP and REV3LF1241A-YFP in PCNA foci relative to background intensities (Image J). Data is from a minimum of 50 foci taken from 7 to 15 cells with comparable protein expression levels of both YFP and CFP tagged proteins. Student two-tailed unpaired *t*-test: (**a**) REV3L and REV3L F1241A-YFP and HcRed-PCNA (*** *p* = 0.0002); (**b**) REV3L and REV3L F1241A-YFP, HcRed-PCNA and KFVLK-CFP (*** *p* < 0.0001). Dotted line represents the value of REV3L-YFP foci intensity without peptide overexpression from (**a**). (**c**) REV3L and REV3L F1241A-YFF, HcRed-PCNA and RWLVK-CFP (** *p* = 0.0011). Dotted line represents the value of REV3L-YFP foci intensity without peptide overexpression from (**a**).

In order to visually detect reduced colocalization/affinity, a large change is required. Fluorescence resonance energy transfer (FRET) measurements would have been the preferred technique, as it can quantifiably differentiate between direct interaction (<20 nm distance) and colocalization (~50–100 nm distance). For example, in a previous study, we found that the direct Xeroderma pigmentosum group-A complementing protein (XPA)–PCNA interaction determined by FRET was abolished when APIM in XPA was mutated; however, XPA with mutated APIM still colocalized with PCNA in replication foci [25]. We have made several attempts to measure FRET between REV3L-YFP (WT and F1241A) and CFP-PCNA. However, the large size of REV3L results in low expression levels and; therefore, low fluorescence intensity compared to PCNA. Therefore, FRET measurements were not technically possible. Second best to FRET, we have measured fold increase in intensity in foci over the background of REV3L-YFP (WT and F1241A), in the absence and presence of overexpressed APIM-CFP. When selecting images taken with the same confocal settings and comparing only cells with equal protein expression levels, we detected higher intensity in foci of REV3L than REV3L F1241A-YFP (Figure 3a). The foci intensities of both full-length proteins were reduced upon overexpression of the APIM motif in REV3L (KFVLK) and importantly, we detected a larger (>2×) reduction for REV3L F1241A than REV3L (26% versus 11%, Figure 3b), suggesting that REV3L F1241A has lower affinity for PCNA than REV3L.

When the high affinity APIM variant (RWLVK) was overexpressed, it nearly abolished the foci formation of both REV3L and REV3L F1241A (Figure 3c). APIM and PIP-box motifs have overlapping interaction sites on PCNA [6,9], and different PIP-box variants are shown to have up to 700× differences in their affinities for PCNA [23], with the PIP-box from p21 being the strongest. The high affinity APIM variant (RWLVK), which has a ~5× lower dissociation constant than the p21 PIP-box in microscale thermophoresis (MST) experiments [17], may be able to compete with both the PIP-box in POLD3 and the APIM in REV3L, for binding to PCNA, explaining the strong reduction of REV3L foci localization observed. We could not detect any differences in colocalization and/or intensities of REV3L in PCNA foci after treatments with DNA inducing damaging agents such as cisplatin, MMC, or UV irradiation (data not shown), although a stronger nuclear localization of REV3L was detected. In summary, these results suggest that REV3L is present in unperturbed replication foci and that the REV3L APIM–PCNA interaction is important for its affinity for PCNA.

### 2.3. Mutation of APIM in REV3L Affects the Mutation Frequency

**Figure 4:**
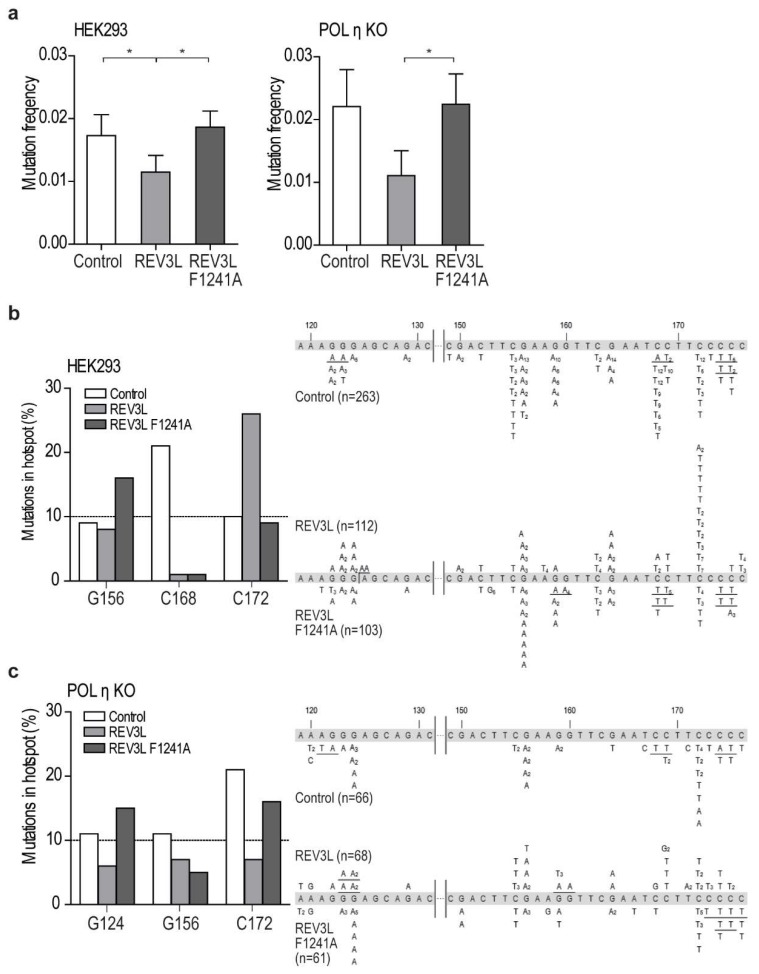
Mutation in APIM in REV3L (F1241A) affects the mutation frequency and pattern. (**a**) Mutation frequency determined by the supF assay from overexpression of REV3L-YFP or REV3L F1241A-YFP in HEK 293 and POLη KO (HAP-1) cells. Cells expressing only UVB-irradiated pSP189 (supF reporter plasmid) represents the control. Four independent experiments are shown for each cell line. Students two-tailed paired *t*-test, * *p* < 0.05. (**b**,**c**) Mutation spectra (*supF* gene) isolated from cells overexpressing REV3L-YFP, REV3L F1241A-YFP, or only pSP189 (control) in HEK293 cells (**b**) and POLη KO cells (**c**) from four independent experiments. Left panel: Mutations at sites in the *supF* gene occurring with a frequency > 10% in either control (white bars), REV3L (light grey bars), or REV3L F1241A-expressing cells (dark grey bars). Right panel: Mutation spectra. The number in subscript indicates how many times the specific mutation was detected in the same transformation. Tandem and quadruple mutations are underlined.

Biochemical assays to test APIM functionality in REV3L is very difficult because REV3L is a very large protein (3130 aa) in complex with multiple other proteins, and interactions with PCNA is a process which is tightly regulated via, for example, PTMs. Therefore, in order to investigate APIM functionality in REV3L, REV3L-YFP (WT and F1241A) were overexpressed in one repair-proficient (HEK293) and one TLS-deficient cell line (POLη KO), together with an UV-irradiated reporter plasmid (SupF mutagenesis assay). The transfection efficiency was 30–50%, and no differences were detected between expression of REV3L and REV3L F1241A (Supplementary Figure S2a). The difference in mutation frequencies between independent experiments detected in the SupF assays was large, still we repeatedly detected a 2–3 times reduction in mutation frequency in cells overexpressing REV3L, compared to cells overexpressing REV3L F1241A, in both cell lines (Figure 4a). Differences in mutation frequencies after REV3L and REV3L F1241A overexpression were also found in two DNA repair-deficient cell lines; a nucleotide excision repair (NER) deficient fibroblast cell line (XPA^−/−^) and a mismatch repair (MMR) deficient cell line (MLH1^−/−^) (Supplementary Figure S2b). Overexpression of REV3L in HEK293 and POLη KO cells reduced the mutation frequency compared to both the control and the REV3L F1241A expressing cells, and this could suggest that APIM in REV3L, and; thus, a direct REV3L–PCNA interaction, contributes to the correct bypass of UV-lesions. Human POLζ has previously been reported to perform error-free replicative bypass of (6-4) photoproducts [26]. However, whether REV3L can bypass lesions correctly or not likely depends upon the damage type and load, and, the DNA repair capacity of the cells. Human POLζ is reported to be able to replicate over UV lesions independently of REV1 [16]. Whether the APIM-mediated PCNA interaction in POLζ is more important for REV1 independent than REV1-dependent bypass of DNA lesions is not possible to predict from our data, and requires additional studies beyond the scope of this paper.

### 2.4. Mutation of APIM in REV3L Affects the Mutation Spectra in Four Cell Lines

The mutation spectra of the *supF* gene isolated from REV3L and REV3L F1241A overexpressing HEK293 cells showed a clear reduction of mutations at position 168 compared to the control cells (Figure 4b, left panel). This verifies that both proteins were expressed in sufficient levels to affect TLS. The spectra importantly also revealed differences between REV3L and REV3L F1241A overexpression (e.g., in the frequency of mutations at position 172 and 156). This suggests that the mutation of APIM in REV3L affected the specificity/function of POLζ.

The mutation spectra from the POLη-deficient cells also showed clear differences between cells overexpressing REV3L and REV3L F1241A at multiple positions, further supporting that APIM in REV3L is important for POLζ’s specificity (Figure 4c). Additionally, and importantly in this context, the mutation spectra from cells overexpressing REV3L and REV3L F1241A were different also in the XPA^−/−^ and MLH1^−/−^ cell lines (Supplementary Figure S2c,d).

Because of the large size of POLζ, previous studies on the polymerase functionality have been done on yeast protein [15] or truncated human REV3L variants [10,14]. This is the first study of the functionality of full-length proteins including the APIM-containing PCD region in REV3L. The differences detected in both the mutation frequencies and the mutation spectra after overexpression of REV3L and REV3L F1241A, in all four cell lines tested, further suggest that APIM in REV3L is a functional PCNA interacting motif. The F1241A mutation is not expected to change the REV3L binding to REV7 nor REV3L’s catalytic activity, because both the REV7 binding region and the catalytic domain are located distant to the PCD region [15]. We hypothesize that the changes in mutation frequency and mutation spectra observed by mutating APIM is due to reduced direct interaction between REV3L and PCNA. Impairing the direct interaction between REV3L and PCNA could either slightly change the proximity of REV3L to DNA, or the switch between the inserter and the extender TLS polymerase, and this could affect TLS and give rise to the differences in mutation frequency and mutation spectra observed between REV3L and REV3F1241A.

POLη interacts with PCNA via the two modules, PIP-box and ubiquitin-binding zinc-finger domain (UBZ), and mutations of one of these modules only partly reduce POLη’s ability to complement Xeroderma pigmentosum variant (XP-V) cells after UV-irradiation [27]. Thus, multiple PCNA interacting modules working cooperatively to stabilize interaction of TLS POLs with PCNA in vivo is known also for other TLS polymerases. Recent data additionally suggests POLη travels with unperturbed replication forks [28]. If TLS polymerases are following a “piggyback” model as suggested (reviewed in [29]) (i.e., they ride on the PCNA ring until the replicative polymerases encounter a DNA lesion), then several interaction motifs of the functional polymerase complex might be required for regulation of their activities.

### 2.5. Targeting PCNA with APIM-Containing Peptides Reduce the Mutation Frequency

**Figure 5:**
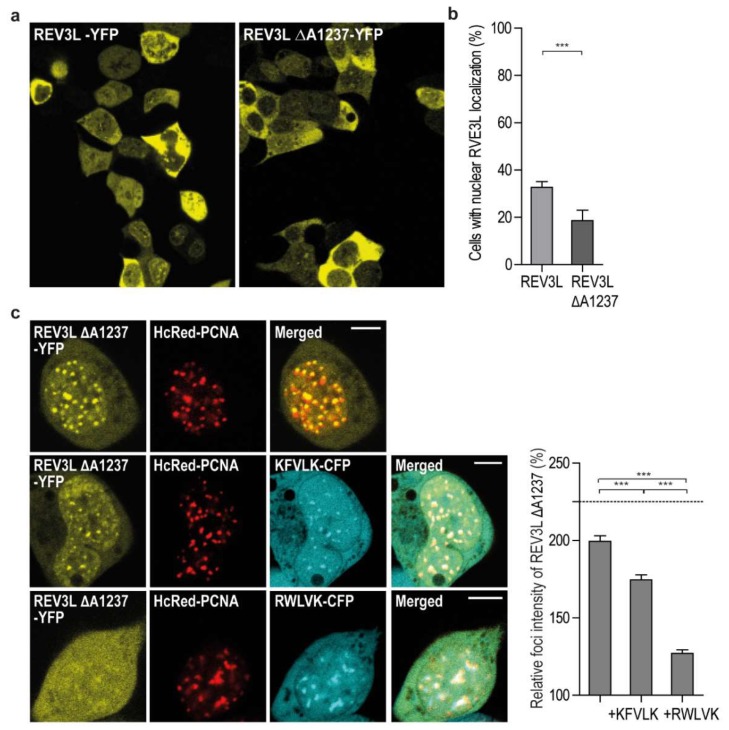
Deletion of A1237 in REV3L affects nuclear localization**.** (**a**) Overview of cells expressing REV3L-YFP and REV3L ΔA1237-YFP in HEK293T cells (live cell images). (**b**) Quantification of the cells with nuclear localization of REV3L-YFP (also shown in Figure 1b) compared to REV3L ΔA1237-YFP. Data from three independent biological experiments from a minimum of 150 cells. (Student *t*-test, *** *p* < 0.0001). (**c**) Left panel: Representative confocal images of HEK293T cells overexpressing REV3L ΔA1237-YFP and HcRed-PCNA (upper row); REV3L ΔA1237–YFP, HcRed-PCNA, and KFVLK-CFP (mid row); and REV3L ΔA1237-YFP, HcRed-PCNA, and RWLVK-CFP (bottom row). White line on merged images represents 5 µm scale. Right panel: Quantification of foci intensities of REV3L ΔA1237 -YFP in PCNA foci over background intensities (Image J). Data from a minimum of 50 foci taken from a minimum of 7 to 10 cells with comparable protein expression levels of both YFP and CFP tagged proteins. (Student two-tailed unpaired *t*-test, *** *p* ≤ 0.0002). Dotted line represents the value of REV3L foci intensity without peptide overexpression from Figure 1a.

In order to further investigate the potential importance of APIM in REV3L, we wanted to make a cell line with an endogenous mutation in the APIM sequence of REV3L. We were not able to create a guide RNA including APIM in REV3L, and therefore decided to use a guide RNA targeting a sequence upstream of APIM. We initially selected mutated cells based on their hypersensitivity to MMC and UV-irradiation, and normal sensitivity to methyl methanesulfonate (MMS), and, surprisingly, the most sensitive clone obtained contained a homozygote single amino acid deletion, two amino acids upstream of APIM (REV3L ∆A1237) (Supplementary Figure S3a–d). No off-targets of significance could explain the observed phenotype in the REV3L ∆A1237 cell line (Supplementary Figure S3e). Because commercial antibodies against REV3L are not available, we tried to establish a targeted MS/MS method for determination of cellular REV3L levels. The level of endogenous REV3L in HEK293 was, as also found by others [11], low and below the detection limit, thus MS/MS detection was not technically possible (levels not detected in four out of five experiments, data not shown). In order to explore the consequence of the ∆A1237 deletion in REV3L, we overexpressed REV3L ∆A1237 as an YFP fusion (REV3L ∆A1237-YFP) and detected reduced nuclear localization of REV3L ∆A1237-YFP compared to REV3L-YFP (Figure 5a,b). Despite this difference, overexpressed REV3L ∆A1237-YFP still colocalized with PCNA when co-expressed with HcRed-PCNA (Figure 5c). Reduced nuclear localization was not detected for the REV3L F1241A mutant (Supplementary Figure S1d), even though both REV3L ∆A1237 and REV3L F1241A have reduced foci intensities compared to REV3L WT (Figure 5c and Figure 3a, respectively). Overexpression of APIM from REV3L (KFVLK) reduced REV3L ∆A1237-YFP foci less than REV3L F1241A-YPF (13% versus 26%, respectively), but slightly more than REV3L WT (11%) (Figure 5c and Figure 3b), indicating that the reduced nuclear localization caused by the ∆A1237 deletion is not mainly due to its reduced PCNA affinity. The single amino acid deletion does not affect nuclear export of REV3L ∆A1237, as Leptomycin B treatment still increased nuclear fraction (data not shown). Thus, reduced nuclear localization and/or stability of REV3L ∆A1237 compared to REV3L WT is likely the main reason for the observed hypersensitivity towards UV-irradiation and MMC in this cell line.

**Figure 6:**
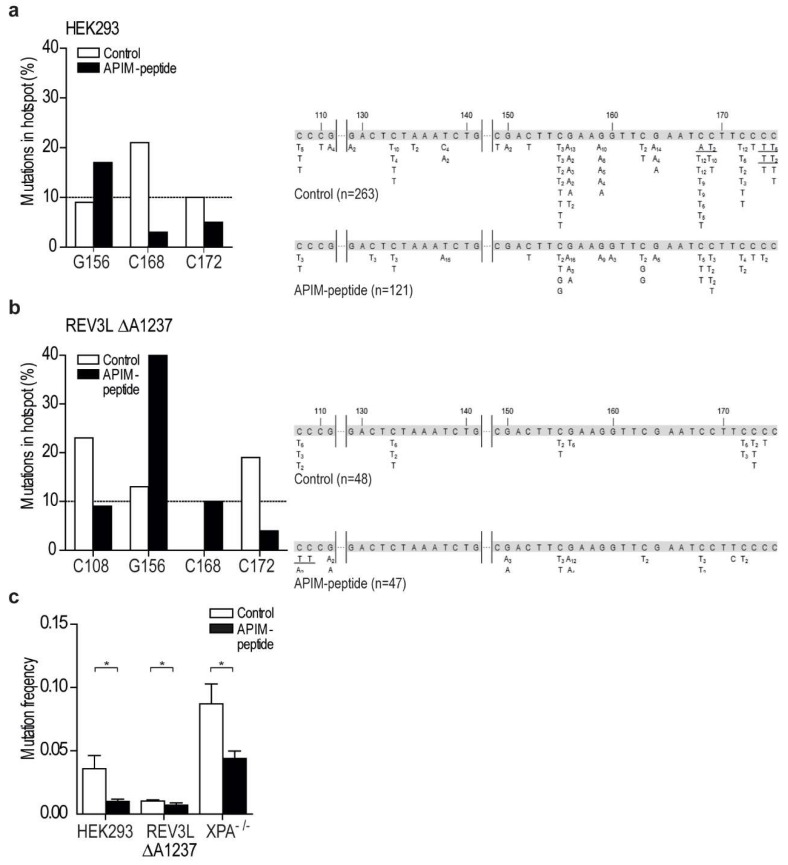
Treatment with APIM-peptide modulates the mutation spectra and reduces the mutation frequencies. (**a**,**b**) Mutation spectra (*supF* gene) from cells transfected with UVB irradiated pSP189 (supF reporter plasmid), with and without treatment with APIM-peptide (10 µM), isolated from (**a**) HEK293 and (**b**) REV3L ∆A1237 cells from four independent experiments. Left panel: Mutations at sites in the *supF* gene occurring with a frequency >10% in either control (white bars) or APIM-peptide treated cells (black bars). Right panel: Mutation spectra. The number in subscript indicates how many times the specific mutation was detected in the same transformation. Tandem mutations are underlined. (**c**) Mutation frequency in HEK293, REV3L ΔA1237, and XPA^−/−^ cells after APIM-peptide treatment (black bars, 10 µM in HEK293 and REV3L ΔA1237, and 8 µM in XPA^−/−^) relative to untreated cells (white bars). Data from each cell line includes data from a minimum of three independent experiments. A significant reduction in mutation frequency after addition of APIM-peptide compared to control (untreated) was detected in all three cell lines (student two-tailed paired *t*-test, * *p* < 0.05).

The mutation spectra of the *supF* gene isolated from REV3L ∆A1237 cells and its isogenic control HEK293 were different, supporting a reduced level of REV3L and an altered TLS pattern in the REV3L ∆A1237 cell line (Figure 6b). For example, mutations in position 168 that are frequent in HEK293 cells were absent in REV3L ∆A1237, and mutations in position 108 were found only in REV3L ∆A1237 (white bars, Figure 6a,b, left panel). Cumulatively, these results show that the REV3L ∆A1237 cell line has an altered TLS response, which, together with its hypersensitivity to MMC, might suggest that it is partly POLζ deficient.

We have previously designed a cell penetrating peptide containing the APIM sequence RWLVK (APIM-peptide), with high PCNA affinity, and a mutated version of this peptide (W4A) with ~50% reduced PCNA affinity [6,17]. Co-treatments with the APIM-peptide are shown to increase the efficacy of multiple chemotherapeutic drugs in multiple cancer cells and preclinical animal models [6,30,31,32]. In Figure 3c we showed that overexpression of RWLVK strongly reduced the colocalization between REV3L and PCNA. We have unpublished data indicating that a significant part of the increased growth inhibitory efficacy observed when combining APIM-peptide with cisplatin (another inter-strand crosslinking agent), is mediated via REV3L inhibition, because siRNA knock down of REV3L had less effect in cells overexpressing the APIM-peptide (data not shown). In order to explore the impact of inhibiting the APIM-mediated REV3L–PCNA interaction on TLS, HEK293 and the REV3L ∆A1237 cells were treated with the APIM-peptide during the SupF assay. In agreement with previous results [6,30], this treatment did not inhibit replication and we were able to isolate newly replicated SupF reporter plasmid. Interestingly, APIM-peptide treatment reduced the mutation frequency in HEK293 cells by ~70% compared to the control, while the reduction was only ~30% in the REV3L ∆A1237 cells (Figure 6c, *p* < 0.05). No reduction in the mutation frequency was detected in similar experiments using the mutant APIM-peptide (Supplementary Figure S4a). There is a tendency towards reduced mutation frequency in REV3L ∆A1237 compared to HEK293 cells when no peptide is added (not significant, *p* = 0.08), suggesting lower levels of REV3L, and reduced TLS, in this cell line. Furthermore, the APIM-peptide was >2× more efficient in reducing the mutation frequency in HEK293 than REV3L ∆A1237 cells. The latter suggests that part of the APIM-peptide’s effect in HEK293 cells is the inhibition of POLζ-mediated TLS. The mutation frequency in the XPA^−/−^ cells was, as expected, elevated compared to the repair proficient HEK293 (~2×); however, a ~50% reduction in mutation frequency was still detected in this cell line after treatments with the APIM-peptide (Figure 6c).

### 2.6. Targeting PCNA with APIM-Peptide Affects the Mutation Spectra

The mutation spectra in the *supF* gene, isolated from both HEK293 and the REV3L ∆A1237 cells, were changed by the APIM-peptide treatment. For example, the mutations at position 168 were strongly reduced compared to the control in HEK293 (Figure 6a, left panel), whereas, in the REV3L ∆A1237 cells, mutations at this position were only detected after treatment with the APIM-peptide (Figure 6b). Similar effects of the APIM-peptide were also detected in the two cell lines, for example, the relative amount of mutations at position 156 were increased after treatment with the APIM-peptide in both cell lines. The APIM-peptide treatment reduced the mutation frequency and changed the mutation spectra in both cell lines, but not similarly. Because the main difference in these two cell lines likely is the level of REV3L, we hypothesize that a major part of the APIM-peptide’s effect on TLS is due to inhibition of the REV3L APIM–PCNA interaction. However, in addition to REV3L, the two Rad5 homologs, HLTF and SHPRH, believed to be important in regulation of TLS [33], and XPA, essential for NER [25], also contain APIM [5]. The function of these proteins could; therefore, also be affected by the APIM-peptide treatment. Of note, the reduction in mutation frequencies shows that the APIM-peptide reduces TLS more efficiently than error free DNA repair, and this could be beneficial in cancer therapies if the APIM-peptide were used in combination with chemotherapeutics.

The aim of this study was to examine if APIM in REV3L is a functional PCNA interacting motif. Our data suggests that the APIM sequence in REV3L is a PCNA interacting motif and that mutation of APIM in full-length REV3L changes the functionality and/or specificity of TLS in vivo. Additionally, we find that targeting PCNA with the APIM-peptide reduces mutagenesis, likely by impairing the efficacy of POLζ. Cumulatively, our data suggests that APIM in REV3L is a functional PCNA interacting motif and that direct interaction with PCNA is important for TLS coordination.

## 3. Material and Methods

### 3.1. Expression Constructs

KFVLK-YFP/CFP and KAVLK-YFP were constructed by annealing MDKFVLK and MDKAVLK encoding oligonucleotides (Sigma-Aldrich, Saint Louis, MO, USA) with EcoRI and XhoI overhang. These were cloned into yellow (YFP) and cyan (CFP) variants of green fluorescent protein (GFP) (Clonetech/TaKaRa Bio USA, Montain View, CA, USA), using the pEYFP-N1 or pECFP-N1 plasmids with mutated ATGsimilarly to RWLVK-CFP [5]. pREV3L-3xFLAG was a kind gift from Christine E. Canman, Department of Pharmacology, University of Michigan, USA [34]. A site-specific mutation F1241A was generated in pREV3L3xFLAG using Quick Change II (Agilent Technologies, Santa Clara, CA, USA). The Amp resistance in this plasmid was switched to Kanamycin (Km) using a Km-resistance gene flanked with AatII and FspI from the pUC57 vector. pREV3L-YFP was generated by GenScript (Piscataway, NJ, , USA) by replacement of the 3xFLAG tag with YFP using the pEYFP-N1. CFP-PCNA and HcRed-PCNA has previously been described [5,35]. pSP189 reporter plasmid and *Escherichia coli* strain MBM7070 were a gift from Professor Karlene Cimprich, Department of Chemical and Systems Biology, Stanford University, Stanford, CA, USA [33].

### 3.2. Cell Lines

HEK293, HEK293T, HCT116, and HeLa cells (ATCC: CRL 1573, CRL 11268, CCL-247, and CCL2, respectively) were cultured in Dulbecco´s Modified Eagle Medium (DMEM) (4.5 g/L; Sigma-Aldrich); and HAP1 cells (POLη KO, Horizon Genomics, Cambridge, UK) were cultured in Iscove’s Modified Medium (IMDM) (Sigma Aldrich). Media was supplemented with 10% fetal bovine serum (FBS; Sigma-Aldrich), 2.5 µg/mL Fungizone^®^ Amphotericin B (Gibco, Thermo Fischer Scientific, Waltham, MA, USA), 1 mM L-Glutamin (Sigma-Aldrich), and an antibiotic mixture containing 100 µg/mL penicillin and 100 µg/mL streptromycin (Gibco). The XP-A deficient cell line (XPA^−/−^; Coriell Institute, GM04429) were cultured in Minimum Essential Medium-alpha (MEM-alpha, 4.5 g/L; Gibco) supplemented with 10% FBS, 2.5 µg/mL Fungizone^®^ Amphotericin B, 1 mM L-Glutamin, and 100 µg/mL gentamicin (Gibco). The cells were cultured at 37 °C in a 5% CO_2_-humidified atmosphere.

### 3.3. SupF Assay

The supF mutagenicity assay was performed essentially as previously reported [33]. Briefly, the reporter plasmid pSP189 was irradiated with 600 or 800 mJ/cm^2^ UVB, depending on the cell line, with UV lamp, Vilber Lourmat Bio Spectra V5, 312 nm. Cells were transfected with UVB-irradiated pSP189 (including plasmids not exposed to UVR as controls) and co-transfected with constructs of interest or treated with APIM-peptide. Transfections were performed using X-treme GENE HP transfection reagent according to manufacturer protocol (Roche diagnostics, Basel, Switzerland). Cells were harvested after 48 h for both isolation of plasmid and western analysis and/or confocal analysis. Isolated plasmids were DpnI (NEB) restriction digested to exclude original bacterial plasmids in order to only look at replicated plasmids. Blue/white screening was performed by transformation of the isolated plasmids into *E. coli* MBM7070 cells, followed by plating on indicator X-gal/IPTG/Amp agar plates. Mutation frequency (white/blue colonies) was calculated for the different samples for several transformations. White mutant colonies were picked for re-streak and DNA sequencing of *supF* gene.

### 3.4. Imaging

HEK293T, transfected with pREV3L-3xFLAG, were fixed in paraformaldehyde (2%) and permeabilized in ice-cold methanol for 5 min at −20 °C. The cells were washed in phosphate buffered saline (PBS) and blocked in bovine serum albumin (BSA)-PBS (2%), prior to incubation with primary antibodies against PCNA (ab18197) and FLAG-peptide (α-FLAG, mouse monoclonal, SIGMA F1804) overnight at 4 °C. Samples were washed in PBS and stained with tetramethylrhodamine (TRITC) goat α-rabbit and Alexa fluor 532 goat α-mouse (Life Technologies), and then diluted 1:200 in BSA-PBS (2%) for 1 hour at room temperature (RT). Samples were washed and maintained in PBS. Images were captured on a Leica SP8 stimulated emission depletion (STED) 3X confocal microscope using a 100×/1.4 oil immersion objective, using the 660 and 775 nm lasers.

For immunofluorescence staining and confocal imaging related to supF assay, cells were transfected in parallel with the SupF-assay transfection, with proportional amounts of cells and transfection mix. Cells were fixed with 2% paraformaldehyde, treated with methanol (−20 °C), and incubated overnight at 4 °C with primary antibody (α-FLAG), diluted 1:500 in FBS-PBS. The following day the cells were washed and treated with secondary antibody (goat α-mouse Alexa fluor 532), diluted 1:2000 in FBS-PBS for 45 min (37 °C). Images were captured using the Zeiss LSM 510 Meta confocal microscope (argon laser 514 nm and BP 530–600 nm for YFP; 543 nm argon laser and LP 560 for Alexa fluor 532). Live cell imaging was performed using a Zeiss LSM 510 Meta laser scanning microscope equipped with a Plan-Apochromate 63x/1.4 oil immersion objective. YFP, CFP, and HcRed were excited and detected at λ = 514 nm/530–600 nm, λ = 458 nm/470–500 nm, and λ = 543 nm/>615 nm, respectively, using consecutive scans. The thickness of the scanned optical slices was 1 µm.

### 3.5. Fluorescence Measurements and Fluorescence Resonance Energy Transfer (FRET)

FRET was done as previously described [6]. Fluorescence intensities was measured using the imaging processing software, Fiji (ImageJ) version 1.06.2016, and all images were taken with the exact same confocal settings. Average intensities within an area of interest (foci) was measured and divided with average intensity in the nucleus outside foci. We selected and compared only cells with equal fluorescence intensities (YFP and CFP) (i.e., equal levels of expressed proteins), and we selected cells within a narrow region of intensities. A minimum of 50 foci were measured per sample from 7 to 15 cells.

### 3.6. Preparation of Cell Lysates

Exponentially grown HEK293T cells were transfected with p-REV3L-YFP or pYFP using X-tremeGENE HP transfection reagent (Roche diagnostics). The cells were crosslinked in 0.25% formaldehyde for 20 min at room temperature, and harvested as previously described [36]. Briefly, the cell pellet was resuspended in 3× packed cell volume in buffer I: 20 mM, pH 7.8, HEPES-KOH, 100 mM KCl, 1.5 mM MgCl_2_, 0.2 mM EDTA, 20% glycerol, 0.5% NP-40, 1 mM DTT, 1× complete protease inhibitor, and 1x phosphatase-inhibitor cocktails I and II (Sigma-Aldrich). 200 U OmniCleave Endonuclease (Epicenter Technologies, Thane, India) was added to each 100 µL of cell pellet before sonication (Branson Sonifier 250). Residual DNA/RNA in the lysates were digested for 1 h at 37 °C using an endonuclease cocktail of 400 U OmniCleave, 10 U DNase I (Roche diagnostics), 250 U benzonase (Merck, Darmstadt, Germany), 100–300 U micrococcal nuclease (Sigma-Aldrich), and 20 µg RNase (Sigma-Aldrich) per 30 mg protein in the lysate. Digestion was followed by clearance by centrifugation.

### 3.7. Immunoprecipitation

Immunoprecipitations were performed using Dynabeads protein A magnetic beads coupled to polyclonal GFP antibodies (ab290, Abcam, Cambridge, UK), which also recognize YFP and CFP, using the crosslinker, Bis(sulfosuccinimidyl)suberate (BS3), according to the manufacturer’s instructions (Thermo Fisher Scientific). Coupled beads were incubated with cleared lysates, under gentle rotation at 4 °C overnight, and further washed, three times, with 10 mM Tris-HCl, pH 7.5, 100 mM KCl before elution. Immunoprecipitated proteins were eluted in lithium dodecyl sulfate (LDS) loading buffer (Invitrogen, Thermo Fisher Scientific), containing 100 mM DTT, by heating the beads for 10 minutes at 70 °C, and separated briefly on a NuPAGE 3–8% Tris-Acetate protein gel (Invitrogen).

### 3.8. Mass Spectrometry (MS) Analysis

The gel lanes were cut into pieces (~100 mg) and submitted to in-gel tryptic digestion, as described by [37]. Tryptic digests were dried out, resuspended in 0.1% formic acid, and analyzed on an Orbitrap Elite mass spectrometer (Thermo Scientific) coupled to an Easy-nLC 1000 UHPLC system (Thermo Scientific). Peptides were injected into an Acclaim PepMap100 C-18 column (75 μm i.d. × 2 cm, C18, 3 μm, 100 Å, Thermo Scientific) and further separated in an Acclaim PepMap RSLC Nanoviper analytical column (75 μm i.d. × 50 cm, C18, 2 μm, 100 Å, Thermo Scientific). A 120-minute method with a 250 nL/minute flow rate was employed; it started with an 80-minute gradient from 2% to 40% of buffer B (99.9% acetonitrile, 0.1% formic acid; buffer A was 0.1% formic acid in water), then it was increased to 55% of buffer B in 15 min, and then to 100% of buffer B in 15 min, it was then kept at 100% of buffer B for 10 min. The peptides eluting from the column were analyzed in positive-ion mode using data dependent acquisition, using collision-induced dissociation (CID) fragmentation with normalized collision energy 35. Each profile MS scan (*m*/*z* 400–1600) was acquired at a resolution of 120,000 FWHM in the orbitrap, followed by 10 centroid MS/MS scans in the ion trap at rapid scan rate, with an isolation width of 2.0 *m*/*z* and an activation time of 10 ms. A 60-second dynamic exclusion was employed. MS spectra were analyzed using Proteome Discoverer (Thermo Scientific) version 1.4.0.288 software, running Mascot and the Sequest HT database search algorithms. Spectra were searched against a human proteome database from UniProt with the following parameters: maximum missed cleavage = 2, precursor mass tolerance = 10 ppm, fragment mass tolerance = 0.6 Da, and dynamic modification = carbamidomethyl (C: +57.021 Da). Peptides were identified with a high degree of confidence (defined as false discovery rate (FDR) ≤0.01) using Percolator. From three biological replicas, possible REV3L interaction partners were identified as proteins detected with a Sequest score >5 in at least two or more experiments in the REV3L-YFP IP, compared to the YFP control sample.

### 3.9. Guide RNA Cloning

LentiCRISPRv2 vector (Addgene plasmid #52961), containing two expression cassettes, hSpCas9 and the chimeric guide RNA, was used as a vector for the CRISPR/Cas9 system. The guide RNA was chosen to target REV3L upstream of its APIM sequence. The following oligonucleotides were used: 5′caccgAAAATCTCAGTCTGGTGCTG-3′ on plus-strand and 5′aaacCAGCACCAGACTGAGATTTTc-3′ on minus-strand. The vector was digested using BsmBI (NEB) and the annealed oligonucleotides (guide RNA) were cloned into the guide RNA scaffold by using Quick Ligase (NEB). Constructs were heat shock transformed into Stbl3 chemically-competent *E. coli*, and plated on LB Ampicillin plates (100 µg/mL). Plasmids from three bacterial colonies were isolated, digested by restriction enzyme HindIII (NEB), and applied on a 0.8% agarose gel for screening. Further, the constructs were sequenced to verify if the guide RNA was cloned correctly into the vector.

### 3.10. Transfection of CRISPR/Cas9 Vector and Single Clone Expansion.

HEK293 cells, seeded in a 12-well plate (150,000 cells/well), were transfected (Xtreme Gene HP, Roche) with 1 µg lentiCRISPR v2 guide plasmid per well. Selection medium containing 2 µg/mL puromycin was added 72 h after transfection and renewed every 3 to 4 days. Potential single-cell colonies could be observed after 14 days. Cell colonies were washed with PBS, trypsinized, resuspended, and transferred into a 24-well plate. Cells were further expanded and DNA was harvested for screening. Briefly, 100,000 cells were resuspended in 50 µL of alkaline solution (25 mM NaOH/0.2 mM EDTA, pH 12) and heated for 10 min at 95 °C. After cooling, 50 µL of neutralizing solution (40mM tris-HCl, pH 5) was added and the lysate was isolated after centrifugation. The target sequence in the REV3L was PCR amplified using forward primer 5′ ATTCTTCTCCACCTCGCTGC-3′ and reverse primer 3′CCGCTATGCACACAATCTGC-5′, and the PCR product was sequenced using forward primer 5′ GCGCAAGAGCACAGATTAAG-3′ and reverse primer 3′ TGGGTAGGGAAGCAGAAAGG-5′.

### 3.11. Viability

HEK293 (4000 cells/well) and REV3L ∆A1237 (5000 cells/well) were seeded in 96-well plates. After 4 h, cells were treated with mitomycin-C (MMC, Medac) (0.05, 0.1, 0.2, and 0.4 µM), exposed to UVB-irradiation (20, 40, and 60 mJ/cm^2^) or treated with methyl methanesulfonate (MMS) (0.025, 0.05, 0.1, and 0.2 µM). Cell viability was measured at different timepoints by the MTT (3-(4.5-Dimethylthiazol-2-yl)-2.5 diphenyl-tetrazolium bromide) assay as previously described [5].

## Supplementary Materials

The following are available online at http://www.mdpi.com/1422-0067/20/1/100/s1, Figure S1 (related to Figure 1): Subcellular localization of YFP and REV3L F1241A-YFP and FRET between PCNA and APIM variants, Figure S2 (related to Figure 3): Mutation frequencies and mutation spectra in XPA and MLH1 deficient cell lines after overexpression of REV3L or REV3L F1241A, Figure S3 (related to Figure 4 and Figure 5): Characterization of the REV3L A1237 cell line, Figure S4 (related to Figure 6): Mutation frequency after treatment with APIM-peptide variants, Table S1 (related to Figure 1): All proteins pulled down with REV3L-YFP used for gene-ontology (GO) analysis.
