# Supplementary material for: APIM-Mediated REV3L–PCNA Interaction Important for Error Free TLS Over UV-Induced DNA Lesions in Human Cells
Source: Int J Mol Sci. 2018 Dec 28;20(1):100. doi: 10.3390/ijms20010100 (PMC6337749; doi:10.3390/ijms20010100)
Supplement: Supplementary file 1 [file ijms-20-00100-s001.pdf]

Supplementary material

APIM mediated REV3L-PCNA interaction important for error free TLS over UV induced DNA lesions in human cells

Synnøve Brandt Ræder[1], Anala Nepal[1,2,#], Karine Øian Bjørås[1,#], Mareike Seelinger[1,#], Rønnaug Steen Kolve[1], Aina Nedal[1], Rebekka Müller[1] and Marit Otterlei[1,2*].

[1]Department of Clinical and Molecular Medicine, Faculty of Medicine and Health Sciences, Norwegian University of Science and Technology (NTNU), Trondheim, Norway

[2]Clinic of Surgery, St. Olavs Hospital, Trondheim University Hospital, Trondheim, Norway

[#]Equal contributions

Supplementary Figure S1

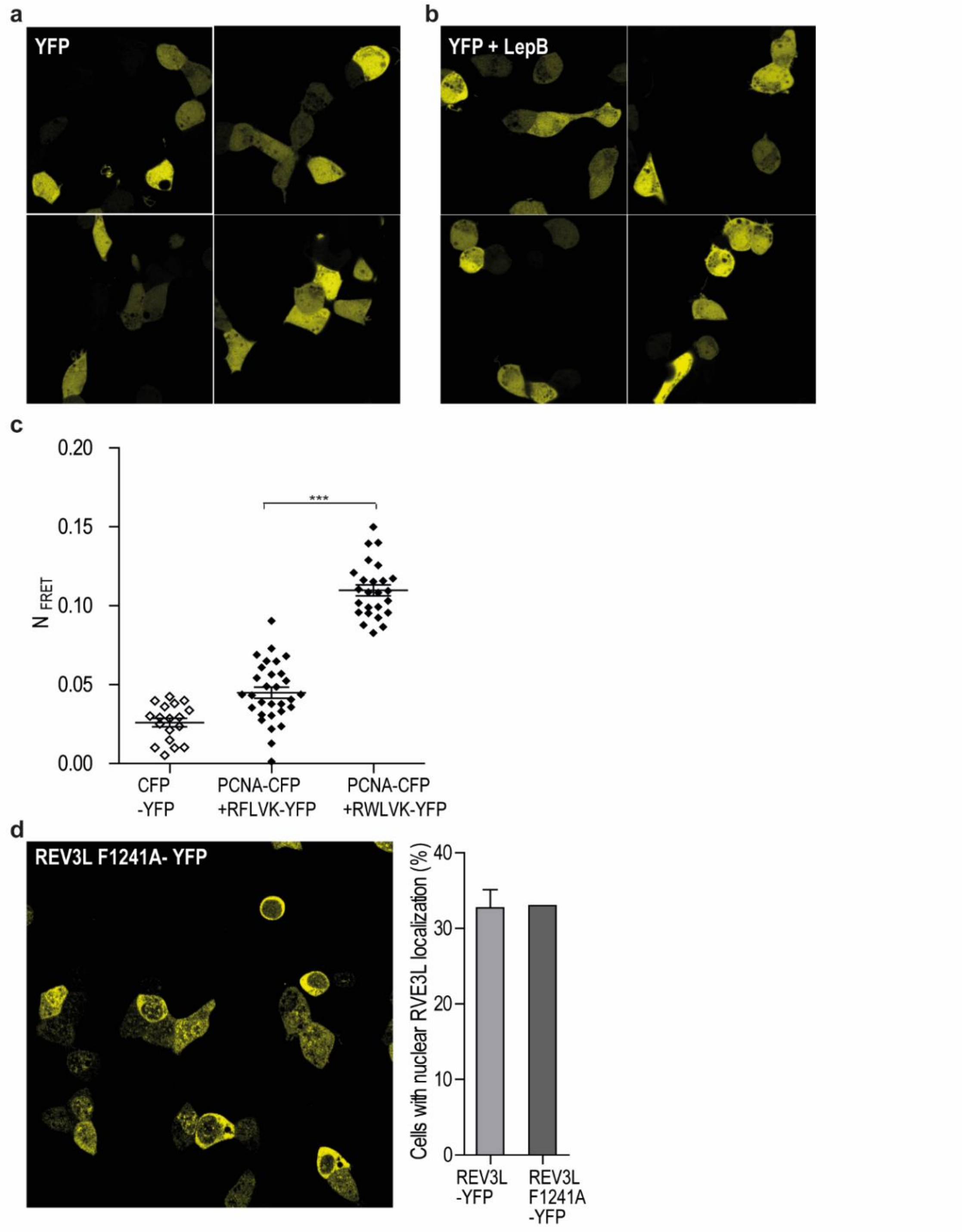

Supplementary Figure S1, related to Figure 1. Subcellular localization of YFP and REV3L F1241-YFP and FRET between PCNA and APIM variants. **(a)** Overview of subcellular localization of YFP expressed in HEK293T cells. **(b)** Overview of subcellular localization of YFP expressed in HEK293T cells with Leptomycin B treatment (10 ng/ml, after 1 hour treatment). **(c)** Normalized FRET measurement between overexpressed PCNA-CFP and RFLVK-YFP or RWLVK-YFP in Hela cells. CFP-YFP (vectors only) was used as background control. Data is from one out of 2 similar experiments. The p-value (***= p> 0.0001) is calculated from a two-tailed unpaired t-test. **(d)** *Left panel:* Overview of subcellular localization of REV3L F1241A- YFP in HEK293T cells. *Right panel:* Quantification of REV3L F1241A-YFP nuclear localization (dark grey bar) from one experiment with in total 267

cells, compared with the quantified REV3L-YFP nuclear localization (light grey bar) also shown previously in Fig. 1b and 5b.

Supplementary Table S1, related to Figure 1. All proteins pulled down with REV3L-YFP used for gene ontology (GO) analysis. **(a)** Proteins immonoprecipitatated from REV3L-YFP expressing cells. Only proteins detected in maximum one out of three IPs from similarly crosslinked YFP-expressing cell are included. Only proteins detected with average sequest score larger than 5 is regarded as a significant detection. Proteins found in the CRAPome database at a frequency of more than 50% of experiments with magnetic Dynabeads are shown in *italic*. These are excluded from the list prior to the GO analysis shown in Fig. 1c. **(b)** GO-analysis of complete list shown in (a) using a PANTHER overrepresentation test. GO biological processes with a Benferroni corrected P-value <0.05 are shown. P-values given as false discovery rate (FDR).

a

Uniprot ID	Symbol	Description	Average sequest score EYFP	REV3L	#Replica detected EYFP	REV3L	CRAPome frequency occurance (%)
P52292	*KPNA2*	*Importin subunit alpha-1*	*4*	*36*	*0*	*3*	*63*
Q03252	*LMNB2*	*Lamin-B2*	*3*	*13*	*0*	*3*	*58*
P49005	POLD2	DNA polymerase delta subunit 2		17	0	3	5
Q15054	POLD3	DNA polymerase delta subunit 3		11	0	3	2,6
O60884	DNAJA2	DnaJ homolog subfamily A member 2	3	11	0	3	50
P34932	HSPA4	Heat shock 70 kDa protein 4	3	8	0	3	39
O60673	DPOLZ	DNA polymerase zeta catalytic subunit	6	671	1	3	0
P45880	*VDAC2*	*Voltage-dependent anion-selective channel protein 2*	*7*	*14*	*1*	*3*	*58*
Q9Y265	*RUVB1*	*RuvB-like 1*	*7*	*29*	*1*	*3*	*66*
O00410	IPO5	Importin-5	7	31	1	3	31,6
Q9Y230	*RUVB2*	*RuvB-like 2*	*7*	*28*	*1*	*3*	*58*
P02545	*LMNA*	*Prelamin-A/C*	*7*	*22*	*1*	*3*	*71*
P55060	CSE1L	Exportin-2	14	30	1	3	45
Q9Y2L1	DIS3	Exosome complex exonuclease RRP44	11	15	1	3	13
O75694	NUP155	Nuclear pore complex protein Nup155	4	13	1	3	42
Q00341	HDLBP	Vigilin	4	8	1	3	18
Q14204	*DYNC1H1*	*Cytoplasmic dynein 1 heavy chain 1*	*12*	*22*	*1*	*3*	*53*
P62906	*RPL10A*	*60S ribosomal protein L10a*	*3*	*5*	*0*	*2*	*58*
P46060	RANGAP1	Ran GTPase-activating protein 1	3	6	0	2	45
P31689	DNAJA1	DnaJ homolog subfamily A member 1	9	14	0	2	50
Q13765	NACA	Nascent polypeptide-associated complex subunit alpha	7	6	0	2	45
O95373	IPO7	Importin-7	14	37	0	2	26
P33992	*MCM5*	*DNA replication licensing factor MCM5*	*3*	*6*	*0*	*2*	*55*
P49588	AARS	Alanine--tRNA ligase, cytoplasmic	4	9	0	2	29
Q16531	DDB1	DNA damage-binding protein 1		7	0	2	50
Q8IX12	CCAR1	Cell division cycle and apoptosis regulator protein 1	2	6	0	2	26
Q86VP6	CAND1	Cullin-associated NEDD8-dissociated protein 1	3	6	0	2	45
Q9Y5V3	MAGED1	Melanoma-associated antigen D1		6	0	2	47
Q6Y7W6	GIGYF2	PERQ amino acid-rich with GYF domain-containing prot 2	3	8	0	2	13
Q92598	HSPH1	Heat shock protein 105 kDa	3	5	0	2	26
Q92616	GCN1L1	Translational activator GCN1	2	12	0	2	34

b

GO biological process	Detected proteins	FDR
Protein import into nucleus	KPNA2, LMNA, NUP155, IPO7, IPO5, CSE1L	3,13E-05
Response to unfolded protein	LMNA, HSPA4, DNAJA1, HSPH1	4,56E-02
DNA-dependent DNA replication	MCM5, POLD2, POLD3, REV3L	3,00E-02
Nucleotide excision repair, DNA incision, 5'- to lesion	POLD2, POLD3, DDB1	2,99E-02
Translesion synthesis	POLD2, POLD3, REV3L	2,85E-02
DNA damage response, detection of DNA damage	POLD2, POLD3, DDB1	2,78E-02

Supplementary Figure S2

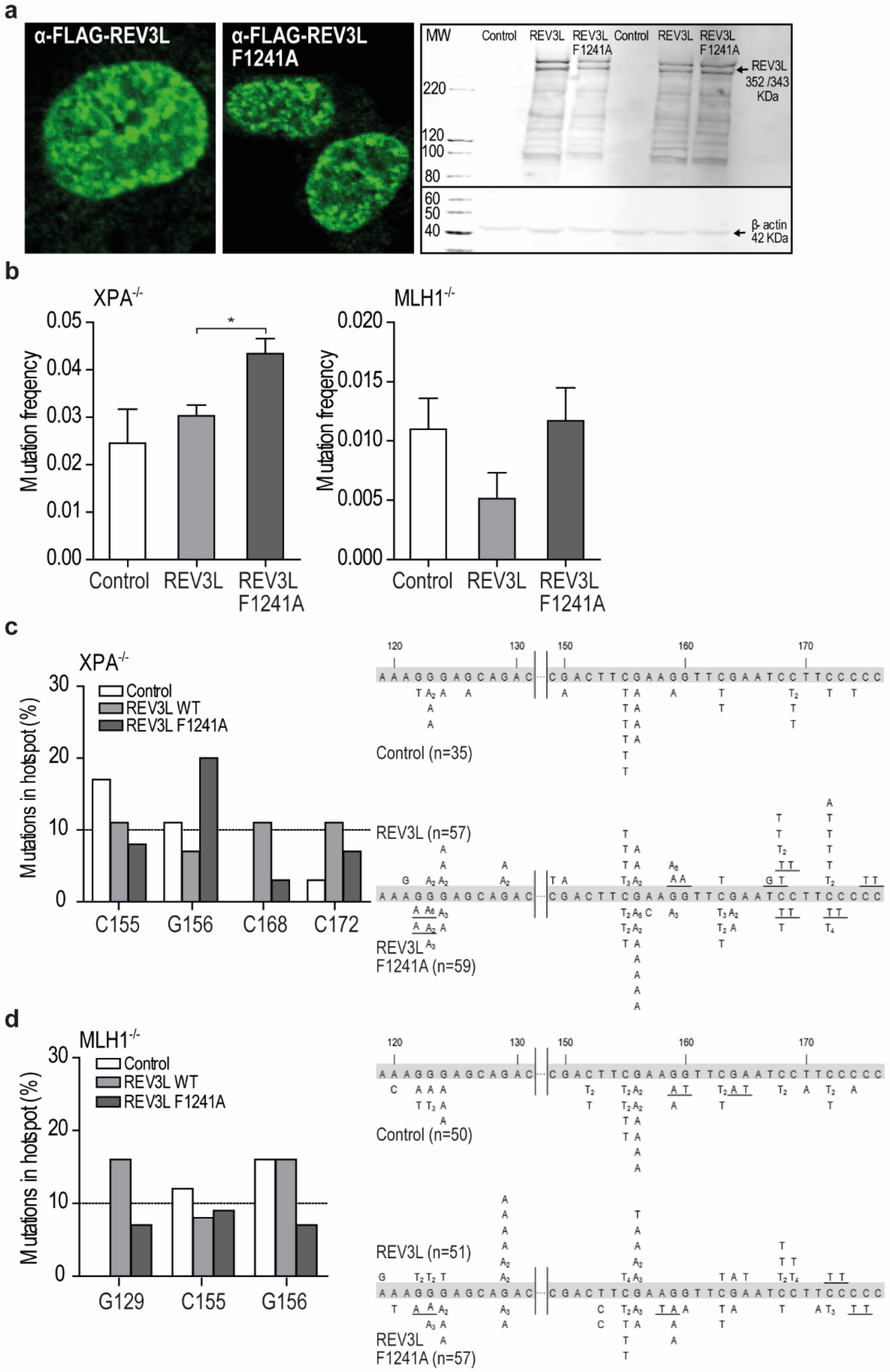

Supplementary Figure S2, related to Figure 3. Mutation frequencies and mutation spectra in XPA and MLH1 deficient cell lines after overexpression of REV3L or REV3L F1241A. **(a)** *Left panel:* Determination of expression levels of FLAG-tagged REV3L and REV3L F1241A by immunofluorescence in HCT116 cells. *Right panel:* Western blot showing expression of FLAG-tagged REV3L and REV3L F1241A in two representative independent experiments (transfections) in HEK293T cells. At least six transfections in HEK293T, HCT116 or XPA$^{-/-}$ cell lines

showed similar results. Western blot analysis was performed as previously described (Olaisen et al., 2015, *Cellular signalling*. 27, (7), 1478-87). Bands below REV3L indicate some degradation of the overexpressed constructs, as these are not detected in the control. **(b)** Mutation frequency determined by SupF assay in XPA $^{-/-}$ (NER deficient) and MLH1 $^{-/-}$ (HCT116, MMR deficient) cells. REV3L WT-FLAG, REV3L F1241A-FLAG or YFP were co-transfected with the UV irradiated reporter plasmid pSP189 (600 and 800 mJ/cm^2 respectively). Cells expressing YFP represents the control. Minimum three independent experiments are shown for each cell line. Students two-tailed paired t-test were performed, *=p<0.05. (**c-d**) Mutation spectra (*supF* gene) isolated from cells overexpressing REV3L-FLAG, REV3L F1241A-FLAG or YFP (control) in (c) XPA $^{-/-}$ cells and (d) MLH1 $^{-/-}$ cells from three independent experiments. *Left panel*: Mutations at sites from the *supF* gene occurring with a frequency >10% in either control-(white bars), REV3L- (light grey bars) or REV3L F1241A-expressing (dark grey bars) cells. *Right panel:* Mutation spectra. The number in subscript indicates how many times the specific mutation was detected in the same transformation. Tandem mutations are underlined.

Supplementary Figure S3

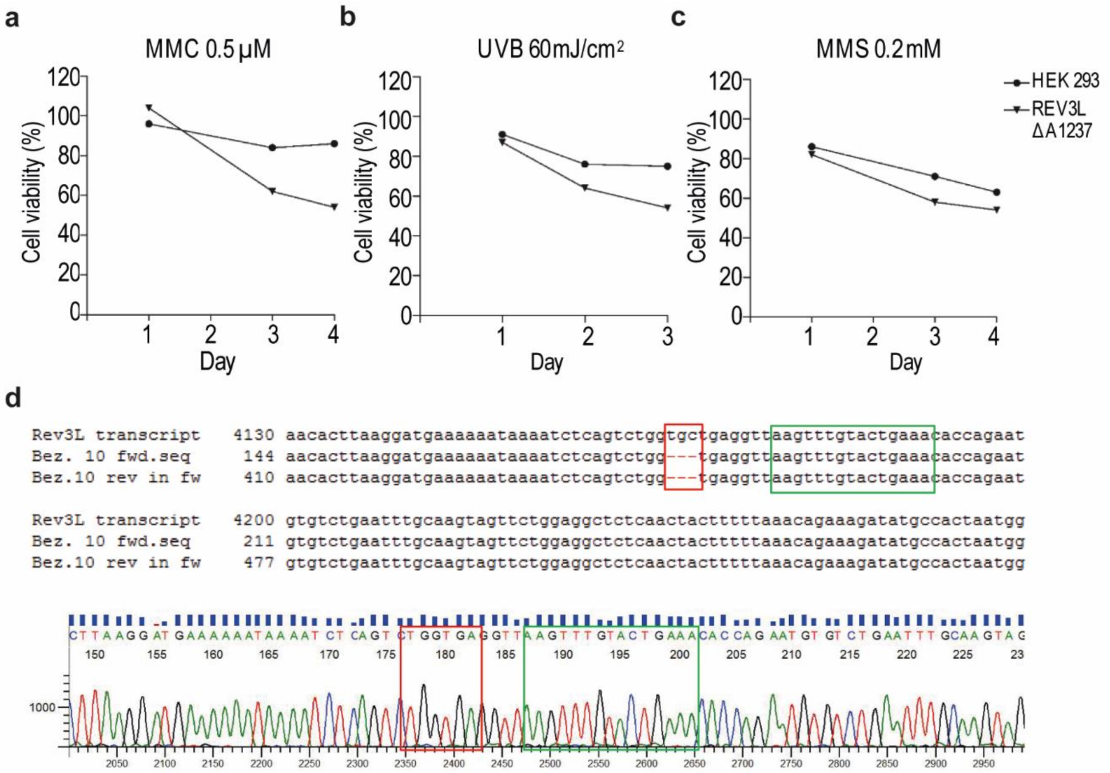

e

	Sequence	Score	Mismatches	Locus
1	AAAATCTCCTTCTGGTGCTGCAG	2.9	2MMs	chr1:-80436663
2	AGAAGCTCATTCTGGTGCTGCAG	2.5	3MMs	chr2:-23273323
3	AAATTCTCAGTCTGGGGCTGTGG	1.7	2MMs	chr1:-68215433
4	AACAACTCAGGCTGGTGCTGCAG	1.5	3MMs	chr4:-25468861
5	AAACTCTCATCCTGGTGCTGAAG	1.3	3MMs	chr11:-115616141
6	AAGAACTCAGTCAGGTGCTGGAG	1.1	3MMs	chr4:+177664033
7	GAAATCACAGCCTGGTGCTGCAG	1.1	3MMs	chr15:-25471587
8	AGAATCCCAGTCTGGTGCTACAG	1.0	3MMs	chr4:-96887131
9	AAACTCTCACTCAGGTGCTGCAG	1.0	3MMs	chr19:+15028070
10	GAAATCTCTGCCTGGTGCTGGAG	1.0	3MMs	chr4:-151344852

Supplementary Figure S3, related to Figure 4 and 5. Characterization of the REV3L ΔA1237 cell line. **(a-c)** Viability of HEK293 and REV3L ΔA1237 after different treatments (treated on day 0)**.** Cells treated with (a)

mitomycin C (MMC), 0.5 µM (b) UVB-irradiation (UVB), 60 mJ/cm^2 and (c) methyl methanesulfonate (MMS), 0.2 mM. One representative experiments out of three independent experiments is presented. **(d)** Confirmation of the deletion of A1237 in REV3L by genome sequencing. *Upper panel:* Genomic REV3L transcript variant 1 isoform gene aligned with REV3L sequenced from REV3L deletion mutant cell line REV3L ΔA1237 (forward and reverse sequence) aligned using software Clone Manager 9. Deleted codon marked with red box, APIM marked with green box. *Lower panel:* Sequence chromatogram from REV3L ΔA1237 investigated in software Sequence Scanner v1.0. Codon triplet upstream and downstream the deleted codon marked with a red box, APIM marked with green box. **(e)** OFF-targets of guide RNA used for mutating genomic REV3L in HEK293 cells with CRISPR/Cas9. Guide RNAsequence including PAM sequence (AAAATCTCAGTCTGGTGCTGAGG) analysed by CRISPR design tool http://crispr.mit.edu/. ON-target chr6:+111695842 (quality score 59), 279-OFF targets found in human genome (18 in genes).

Supplementary Figure S4

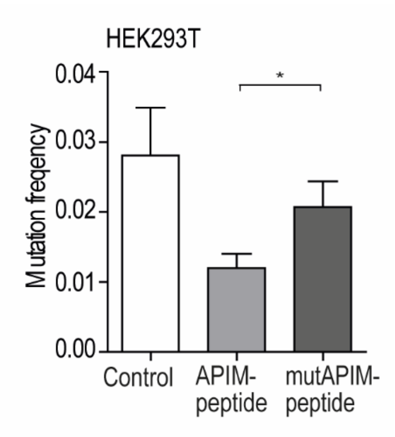

Supplementary Figure S4, related to Figure 6. Mutation frequency after treatment with APIM-peptide variants. APIM-peptide, but not the mutAPIM-peptide, reduced the mutation frequency in HEK293T. Mutation frequency determined by the SupF assay after treatment with APIM-peptide or mutAPIM-peptide (10 µM). Data from four independent experiments. Two-tailed paired t-test (*=p<0.05).
